# Molecular characterization of colistin resistance in carbapenem-resistant *Klebsiella pneumoniae* from a tertiary hospital in China

**DOI:** 10.1128/spectrum.01049-25

**Published:** 2025-08-29

**Authors:** Saiqi Qi, Haijun Li, Jilei Ma, Cailin Liu, Nan Yi, Shijun Sun, Wanhai Wang

**Affiliations:** 1 Department of Clinical Laboratory, The First Affiliated Hospital of Zhengzhou University 191599, Zhengzhou, China; 2 Key Clinical Laboratory of Henan Province, Zhengzhou, China; University at Albany, Albany, New York, USA

**Keywords:** colistin resistance, *mcr*/TCS gene, *mgrB*, insertion sequence, carbapenem-resistant *Klebsiella pneumoniae* (CRKP)

## Abstract

Colistin resistance in carbapenem-resistant *Klebsiella pneumoniae* (CRKP) poses a significant global health challenge, as colistin remains the last-resort antibiotic for treating multidrug-resistant *K. pneumoniae* infections. This study aimed to investigate the prevalence and molecular mechanisms underlying colistin resistance in CRKP (Col^r^-CRKP) isolates in Henan, China, from 2021 to 2024. The minimum inhibitory concentrations of colistin for 134 *K*. *pneumoniae* isolates were determined using the broth microdilution method. Whole-genome sequencing was performed using the Illumina platform to identify carbapenemase genes and sequence types (STs). Colistin resistance mechanisms were investigated, including mutations in two-component systems (*pmrA*/*pmrB*, *phoP*/*phoQ*), inactivation of the *mgrB* gene, and the presence of plasmid-mediated *mcr* genes. Most isolates were collected from intensive care units (99/134, 73.9%), with 48.5% (59/134) of patients having no documented colistin exposure history. Notably, ST11 was the predominant sequence type among Col^r^-CRKP isolates (113/134, 84.3%), all of which carried *bla*_KPC-2_ as the sole carbapenemase determinant. In contrast, seven non-carbapenemase-producing isolates exhibited phenotypic resistance to carbapenems. Genomic analysis revealed inactivation or loss of the *mgrB* gene in 53.7% (72/134) of isolates, predominantly due to insertion mutations (54/72). Although 32.8% (44/134) of isolates carried mutations in two-component systems, these alterations did not exhibit pathway-specific clustering. Intriguingly, plasmid-mediated *mcr* genes were detected in only 1.5% (2/134) of cases (*mcr-8.2* and *mcr-1.1*), while 22.4% (30/134) of colistin-resistant strains lacked identifiable resistance determinants based on current detection methods. Our findings indicate that disruption of the *mgrB* gene is the primary mechanism of colistin resistance in ST11 CRKP clones. The emergence of resistance in 48.5% of patients without prior colistin exposure, combined with low *mcr* gene prevalence (1.5%) and unexplained resistance in 22.4% of isolates, suggests complex selective pressures beyond direct antimicrobial use. These findings underscore the urgent need for strengthened antimicrobial stewardship and the development of alternative therapeutic strategies to combat this high-risk pathogen.

**IMPORTANCE:**

The global rise of colistin-resistant *Klebsiella pneumoniae*, particularly in carbapenem-resistant *Klebsiella pneumoniae* (CRKP) strains, has severely restricted treatment options for multidrug-resistant infections. Our study provides the first comprehensive molecular characterization of colistin resistance in CRKP in a large tertiary hospital in central China. We identified *mgrB* disruption as the predominant resistance mechanism, while plasmid-mediated *mcr* genes were rare. Notably, nearly half of the resistant isolates occurred in patients without prior colistin exposure, suggesting alternative selective pressures driving resistance. These findings highlight the complex dynamics of colistin resistance in CRKP and underscore the need for enhanced genomic surveillance and stewardship interventions to limit further dissemination.

## INTRODUCTION

*Klebsiella pneumoniae*, a member of the Enterobacterales order, is traditionally regarded as an opportunistic pathogen capable of causing urinary tract infections, pneumonia, and bloodstream infections (1). *K. pneumoniae* has garnered increasing attention primarily due to its drug resistance, with carbapenem-resistant *Klebsiella pneumoniae* (CRKP) being the most prevalent form. In recent years, CRKP has rapidly disseminated worldwide and has been designated a critical priority pathogen by the World Health Organization (2). A recent study reported a pooled mortality rate of 37.2% associated with CRKP infections (3). The production of carbapenemases is the predominant resistance mechanism in CRKP (4, 5). In China, CRKP isolates from adults primarily produce *Klebsiella pneumoniae* carbapenemase (KPC), whereas New Delhi metallo-β-lactamase (NDM) is more frequently observed in pediatric cases. The emergence of numerous CRKP strains, coupled with the lack of effective new antibiotics, has become a major global health concern (6).

Polymyxins, including polymyxin B and polymyxin E (colistin), have served as the last line of defense against CRKP. However, the use of colistin declined in the 1980s due to reports of nephrotoxicity and neurotoxicity (7, 8). Colistin is a polycationic peptide that exerts its antimicrobial activity by targeting the negatively charged components of bacterial lipopolysaccharides (LPS), a major constituent of the gram-negative outer membrane (9).

The rising trend of colistin resistance among gram-negative bacteria has attracted global concern, prompting extensive investigation into its underlying mechanisms. Since the initial discovery of the *mcr* gene in *K. pneumoniae*, which mediates lipid A modification, numerous horizontally transferred colistin resistance mechanisms have been described (10, 11). To date, all known *mcr* gene variants have been identified and are widely disseminated across the globe. Among them, *mcr-1*, *mcr-3*, *mcr-7*, *mcr-8*, and *mcr-10* have been reported in *K. pneumoniae* (12). The *mcr* gene confers resistance to colistin by encoding an enzyme that adds phosphoethanolamine (pEtN) to lipid A, thereby reducing membrane affinity to the drug (13). A retrospective study by Shen et al. in China revealed that the *mcr-1* gene could be traced back to the 1980s in *Escherichia coli* isolates from poultry, and the earliest human-associated *mcr*-positive bacterium was a *mcr-1*-harboring strain of *Shigella sonnei* identified in 2008 (14, 15). Among the reported *mcr*-positive isolates, livestock was the most common source, followed by humans, meat, and food products. Additional reservoirs—including pets, wild animals, and environmental sources—have also been documented in 57 countries, with the exception of Antarctica (10).

Additionally, colistin resistance most commonly arises from LPS modifications regulated by two-component systems (TCSs), including *pmrA*/*pmrB*, *phoP*/*phoQ*, and the *mgrB* gene, a negative regulator of the *phoP*/*phoQ* pathway. Briefly, the TCS pathways *pmrA*/*pmrB* and *phoP*/*phoQ* can modify the surface charge of LPS—the initial binding target of colistin—by adding cationic groups such as pEtN and/or 4-amino-4-deoxy-L-arabinose (L-Ara4N) (9). The *mgrB* gene, a 144 bp regulator, not only negatively regulates *phoP*/*phoQ* expression but may also alter the structural characteristics of the bacterial membrane, thereby contributing to colistin resistance (12).

The emergence and spread of colistin resistance in CRKP (Col^r^-CRKP) represent a serious threat to public health, particularly in hospital settings, due to the limited availability of effective treatment options. A nationwide study reported that 43% of KPC-producing *K. pneumoniae* isolates in China were resistant to colistin (16). Alarmingly, multiple outbreaks of carbapenem-resistant and colistin-resistant isolates have been reported in North America and Europe (9).

With the increasing number of reported outbreaks, the threat posed by Col^r^-CRKP is becoming more serious. Timely clinical surveillance and epidemiological data collection are essential for controlling the spread of Col^r^-CRKP and safeguarding public health. Appropriate antibiotic use may reduce the selective pressure for the emergence of Col^r^-CRKP. Moreover, surveillance findings can serve as an early warning for clinicians to prevent widespread transmission. To date, relatively few epidemiological investigations have focused on Col^r^-CRKP, despite the severity of the situation. In this study, we aimed to investigate the prevalence and explore the molecular mechanisms underlying colistin resistance in a collection of CRKP isolates obtained from clinical specimens in Zhengzhou, Henan.

## MATERIALS AND METHODS

### Sample collection and isolation

A total of 134 Col^r^-CRKP isolates were collected from clinical specimens at the First Affiliated Hospital of Zhengzhou University between February 2021 and January 2024. Species identification was confirmed using matrix-assisted laser desorption/ionization time-of-flight mass spectrometry. Clinical metadata, including specimen sources (e.g., respiratory secretions, blood) and hospital collection units, were retrieved from the electronic medical records. All isolates were preserved in Luria–Bertani (LB) broth supplemented with 15% glycerol and stored at −80°C. For experimental use, frozen stocks were thawed and inoculated into LB broth using sterile loops, followed by overnight incubation at 35°C. *Escherichia coli* ATCC 25922 was used as a quality control strain for antimicrobial susceptibility testing.

### Whole-genome sequencing and analysis

Genomic DNA from the 134 isolates was extracted using the TIANamp Genomic DNA Kit (Tiangen Biotech, Beijing, China), and whole-genome sequencing was performed on the Illumina HiSeq X Ten platform. Raw sequencing reads were assessed for quality using FastQC v0.11.9 and trimmed with Trimmomatic v0.39. The FastQ data generated from the Illumina platforms were assembled using SPAdes v4.0.0, and the genome assemblies were annotated with Prokka v1.14.6 (17–19). Multilocus sequence types (STs) and antimicrobial resistance genes were identified using the Center for Genomic Epidemiology database (https://genomicepidemiology.org/services/). All resistance genes were further confirmed using ResFinder (http://genepi.food.dtu.dk/resfinder) and the Basic Local Alignment Search Tool (BLAST).

### Antimicrobial susceptibility testing

Antimicrobial susceptibility of the 134 isolates was determined using the agar dilution (Mueller-Hinton agar, Oxoid, UK) and broth microdilution methods (Mueller-Hinton broth, BD Diagnostics, USA) in accordance with Clinical and Laboratory Standards Institute guidelines (M100-Ed35) (20). Susceptibility of the CRKP isolates to 13 antibiotics—amikacin (AMK), meropenem (MEM), imipenem (IPM), ciprofloxacin (CIP), cefuroxime (CXM), ceftriaxone (CRO), ceftazidime (CAZ), minocycline (MNO), piperacillin/tazobactam (TZP), tigecycline (TGC), tobramycin (TOB), colistin (COL), and levofloxacin (LVX)—was assessed. *Escherichia coli* ATCC 25922 was used for quality control. Colistin minimum inhibitory concentration (MIC) breakpoints were interpreted based on European Committee on Antimicrobial Susceptibility Testing guidelines (https://www.eucast.org/clinical_breakpoints) (21). Antibiotic concentration gradients ranged from 0.12 mg/L to 256 mg/L and were prepared using twofold serial dilution. The susceptibility profiles were visualized using Chiplot (https://www.chiplot.online/upset_plot.html) (22).

### Phylogenetic analysis

Multiple sequence alignments of the 134 isolates were generated using Roary v3.12.0. A concatenated alignment of 3,904 core genes was constructed for phylogenetic reconstruction. An initial maximum-likelihood phylogenetic tree was constructed with RAxML v8.2.12 under the GTRGAMMA model (using 1,000 bootstrap replicates). Recombination events were then identified and removed using ClonalFrameML, with the RAxML-generated tree serving as input. Subsequently, the final clonal genealogy was inferred by re-running RAxML v8.2.12 on the recombination-filtered alignment. The phylogenetic tree was finally visualized and annotated using the Tree Visualization By One Table (TVBOT) online tool (https://www.chiplot.online/tvbot.html) (23).

### Determinants of colistin resistance

Key genes involved in colistin resistance mechanisms (*mgrB*, *pmrA*/*pmrB*, *phoP*/*phoQ*, and *mcr*) were screened using ResFinder (https://cge.cbs.dtu.dk/services/ResFinder/) and BLAST. Insertion sequences within the *mgrB* gene were identified using ISfinder (https://isfinder.biotoul.fr/about.php). The lolliplot function in the TrackViewer package in RStudio (v2024 12.0+) was used to visualize mutation sites in the *mgrB* gene.

### Statistical analysis

To assess the association between resistance mechanisms and clonal lineages, statistical analyses were performed using R software (v4.3.2). The chi-square test or Fisher’s exact test was used, as appropriate, to compare the distribution of colistin resistance mechanisms (e.g., *mgrB* inactivation, TCS mutations, presence of *mcr* genes) across different STs or clone clusters. A *P*-value <0.05 was considered statistically significant. The relationship between specific mutations and resistance phenotypes was also evaluated to identify potential lineage-specific patterns. These analyses aimed to strengthen the validity of our findings and provide a more robust interpretation of resistance dissemination.

## RESULTS

### Clinical isolate collection and characteristics

**Fig 1:**
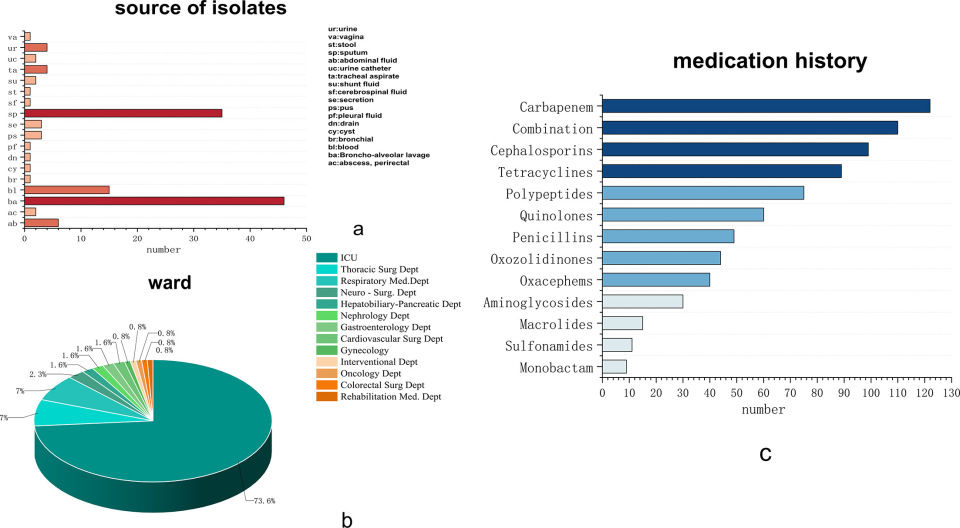
The clinical information of 134 strains. (**a**) The source of isolates. (**b**) Ward information for 134 strains. (**c**) The medication history of patients. Combination: β-lactam/β-lactamase inhibitor combinations.

A total of 140 non-duplicate clinical samples were collected from the First Affiliated Hospital of Zhengzhou University, a leading tertiary Grade-A hospital in China, between February 2021 and January 2024. Following the exclusion of six strains due to contamination or duplication, 134 isolates remained for analysis. The origin of these isolates was primarily the intensive care unit (ICU) (99/134, 73.9%), followed by lung transplant surgery wards (10/134, 7.5%) and respiratory medicine departments (9/134, 6.7%). Isolates from other general wards (e.g., urology, otolaryngology) were infrequent (0.7%–2.2%). The predominant sample types were bronchoalveolar lavage fluid (46/134, 34.3%) and sputum (34/134, 25.3%) (Fig. 1).

**Fig 2:**
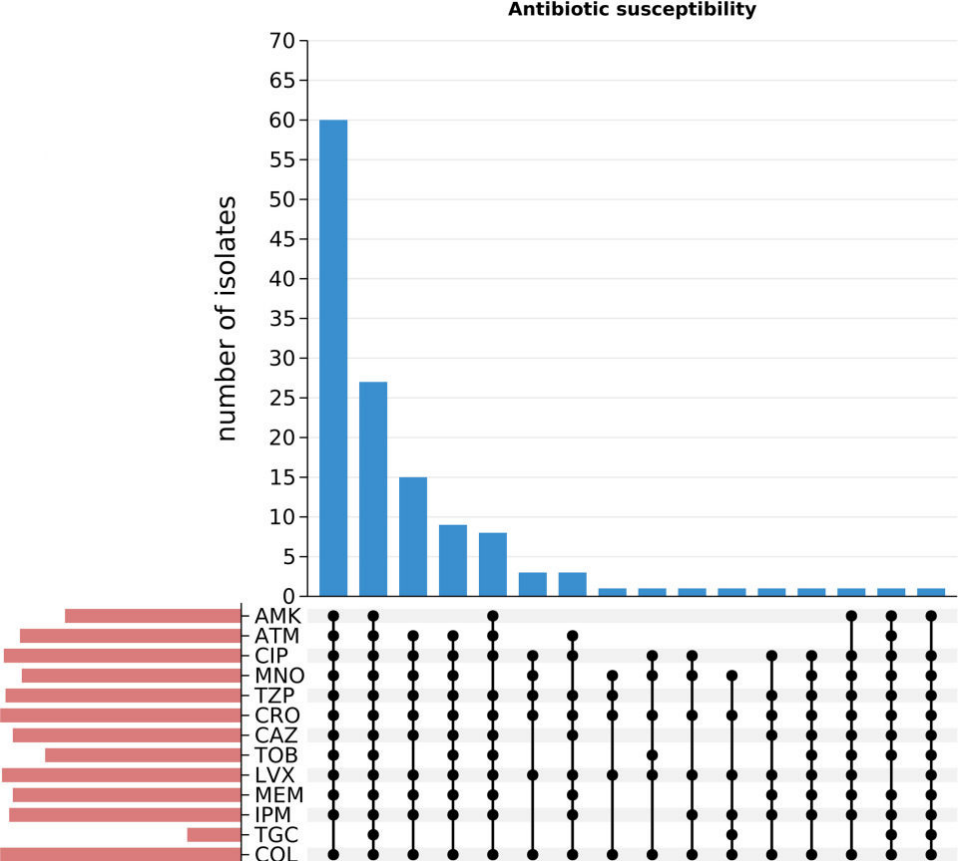
An UpSet plot showing the total number of isolates resistant to antimicrobials tested (left bar plot), the total number of isolates exhibiting a particular antibiogram (top bar plot), and filled dots representing the presence of an antimicrobial resistance phenotype. AMK, amikacin; ATM, aztreonam; CIP, ciprofloxacin; MNO, minocycline; TZP, piperacillin/tazobactam; CRO, ceftriaxone; CAZ, ceftazidime; TOB, tobramycin; LVX, levofloxacin; MEM, meropenem; IPM, imipenem; COL, colistin; and TGC, tigecycline.

Antimicrobial susceptibility profiles are summarized in Fig. 2 (original data are presented in Table S1). Patient medication histories revealed predominant utilization of β-lactam antibiotics, alongside exposure to antimicrobial agents spanning 13 distinct classes: cephalosporins, penicillins, macrolides, carbapenems, tetracyclines, polypeptides, sulfonamides, oxazolidinones, quinolones, aminoglycosides, monobactams, oxacephems, and β-lactam/β-lactamase inhibitor combinations (including piperacillin/tazobactam, mezlocillin/sulbactam, cefoperazone/sulbactam, and ceftazidime/avibactam). The five most frequently administered antibiotic classes were carbapenems (*n* = 122), β-lactam/β-lactamase inhibitor combinations (*n* = 110), cephalosporins (*n* = 99), tetracyclines (*n* = 89), and polypeptides (*n* = 75). Resistance profiling demonstrated that the majority of isolates were resistant to most agents, with susceptibility observed only to tigecycline, cefuroxime, and ampicillin/sulbactam. Furthermore, a subset of strains displayed extensively drug-resistant phenotypes, demonstrating non-susceptibility to all currently recommended first-line therapeutic agents specified in clinical guidelines (Fig. 2).

### Epidemiological distribution of *K. pneumoniae* clones

**Fig 3:**
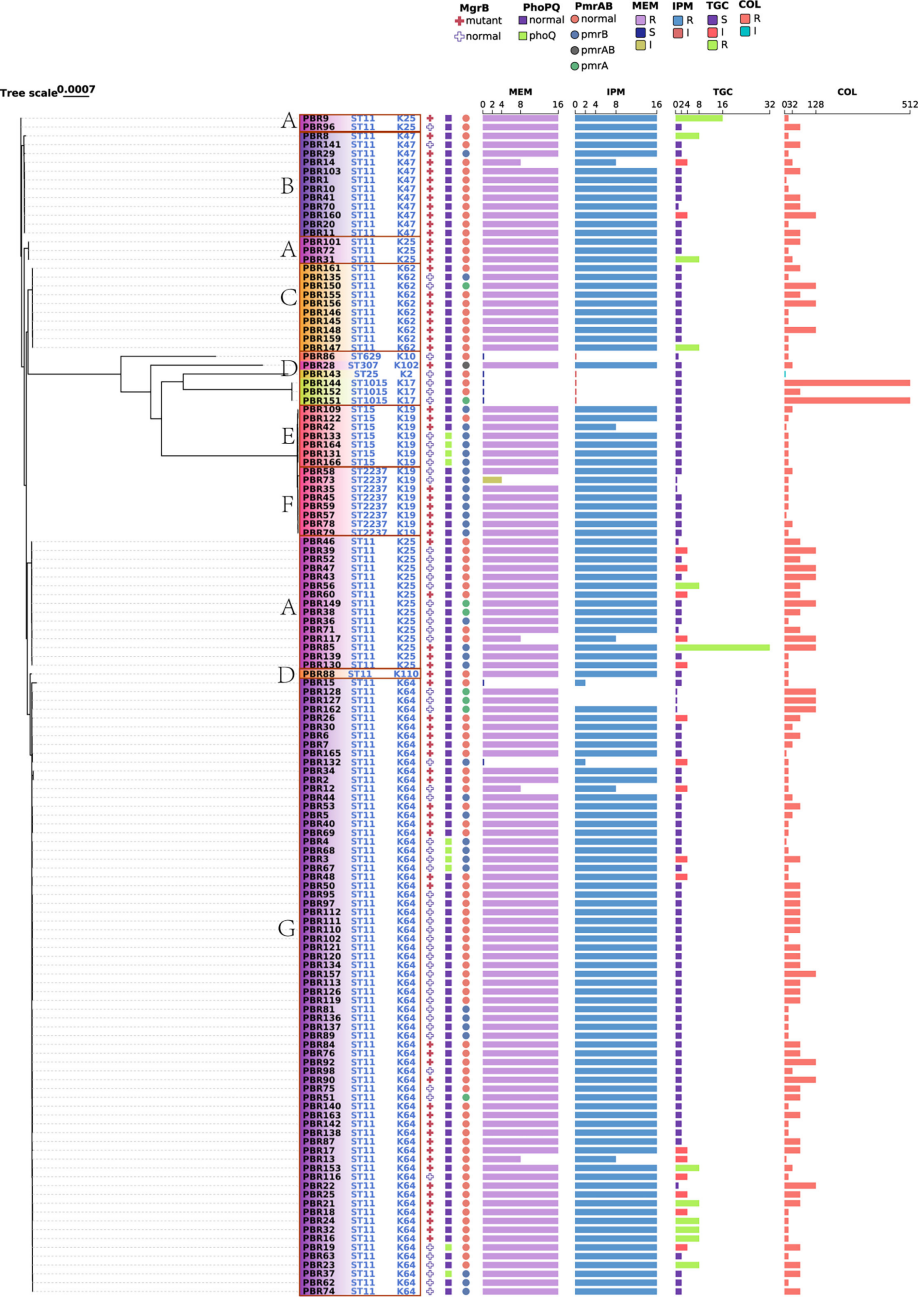
The phylogenetic relationships, mutant gene, and antimicrobial susceptibility profiles of the collected isolates were analyzed. All isolates were grouped into seven clusters according to ST type. To analyze the isolates briefly, we gathered the uncommon type in Cluster D. Mutant gene *mgrB*, *phoP*/*phoQ*, and *pmrA*/*pmrB* are shown in the figure. Abbreviations for antimicrobial agents are as follows: MEM, meropenem; IPM, imipenem; TGC, tigecycline; and COL, colistin. Different color blocks indicate different patterns. R, resistant; I, intermediary; and S, susceptible.

A phylogenetic tree was constructed using RAxML and visualized with TVBOT (Fig. 3). Capsular serotype K64 (52.2%) and sequence type ST11 (84.3%) were the most prevalent, followed by K25 (14.9%), K19 (11.1%), and K47 (8.9%). In addition to the predominant ST11-K64 genotype, we also identified clusters of ST11-K25, ST11-K47, and ST11-K62 strains. To facilitate accurate classification and comparative analysis, isolates were grouped into seven clusters (A–G) based on capsular (K) and sequence (ST) types, with Cluster G (ST11-K64) being the largest. Rare types were consolidated into Cluster D. Cluster-based analysis revealed consistent antimicrobial susceptibility patterns within each group. All isolates exhibited colistin non-susceptibility, while resistance to imipenem (IPM) and meropenem (MEM) exceeded 90%. In contrast, most isolates retained susceptibility to tigecycline (Fig. 3). Colistin resistance mechanisms, including *pmrA*/*pmrB* and *phoP*/*phoQ* mutations and *mgrB* alterations, were mapped onto the phylogenetic tree. Chromosomal *mgrB* disruptions (53.7%) were the predominant mechanism of colistin resistance, followed by *pmrA*/*pmrB* (32.1%) and *phoP*/*phoQ* (7.4%) mutations, as shown in Fig. 3.

### Carbapenemase genotype distribution

Carbapenemase production was the primary resistance mechanism among CRKP isolates. The *bla*_KPC-2_ gene was detected in 93.2% (125/134) of the isolates. Notably, two isolates co-harbored metallo-β-lactamase genes: one carried *bla*_NDM-1_ and the other *bla*_NDM-5_. No carbapenemase genes were detected in the remaining seven isolates (PBR58, PBR73, PBR86, PBR143, PBR144, PBR151, and PBR152), all of which exhibited significantly lower meropenem and imipenem MIC values compared to *bla*_KPC-2_-positive isolates.

### Prevalence of plasmid-mediated *mcr* genes

ResFinder analysis revealed a limited distribution of *mcr* genes among the isolates, with only two positive cases identified. Specifically, the *mcr-1.1* gene was detected in isolate PBR86, while PBR96 harbored the *mcr-8.2* variant. Comprehensive screening confirmed the absence of additional *mcr* gene variants in this collection (Table 1).

**TABLE 1:** Numbers and percentages of colistin-resistant-related gene

Gene	Mutant	Number	Percentage
*pmrB*	–^*a*^	99	73.8
	T157P	16	11.9
	A246T	14	10.4
	730-740del	3	2.2
	M213L	1	0.7
	S203P	1	0.7
*pmrA*	–	125	93.2
	G157T(G53C)	7	5.2
	G121A(A41T)	1	0.7
	Partial deletion	1	0.7
*phoQ*	–	124	92.5
	A466T(T156S)	4	2.9
	G268T(D90Y)	4	2.9
	1-797del	1	0.7
	T365A(I122N)	1	0.7
*mcr*	–	132	98.5
	*mcr-1.1*	1	0.7
	*mcr-8.2*	1	0.7
*mgrB*	–	62	46.2
	Del	7	5.2
	Insert	54	40.2
	Point mutation	11	8.2

^*a*^ "–" represents no mutation in the genes.

### Chromosomal determinants of colistin resistance

**Fig 4:**
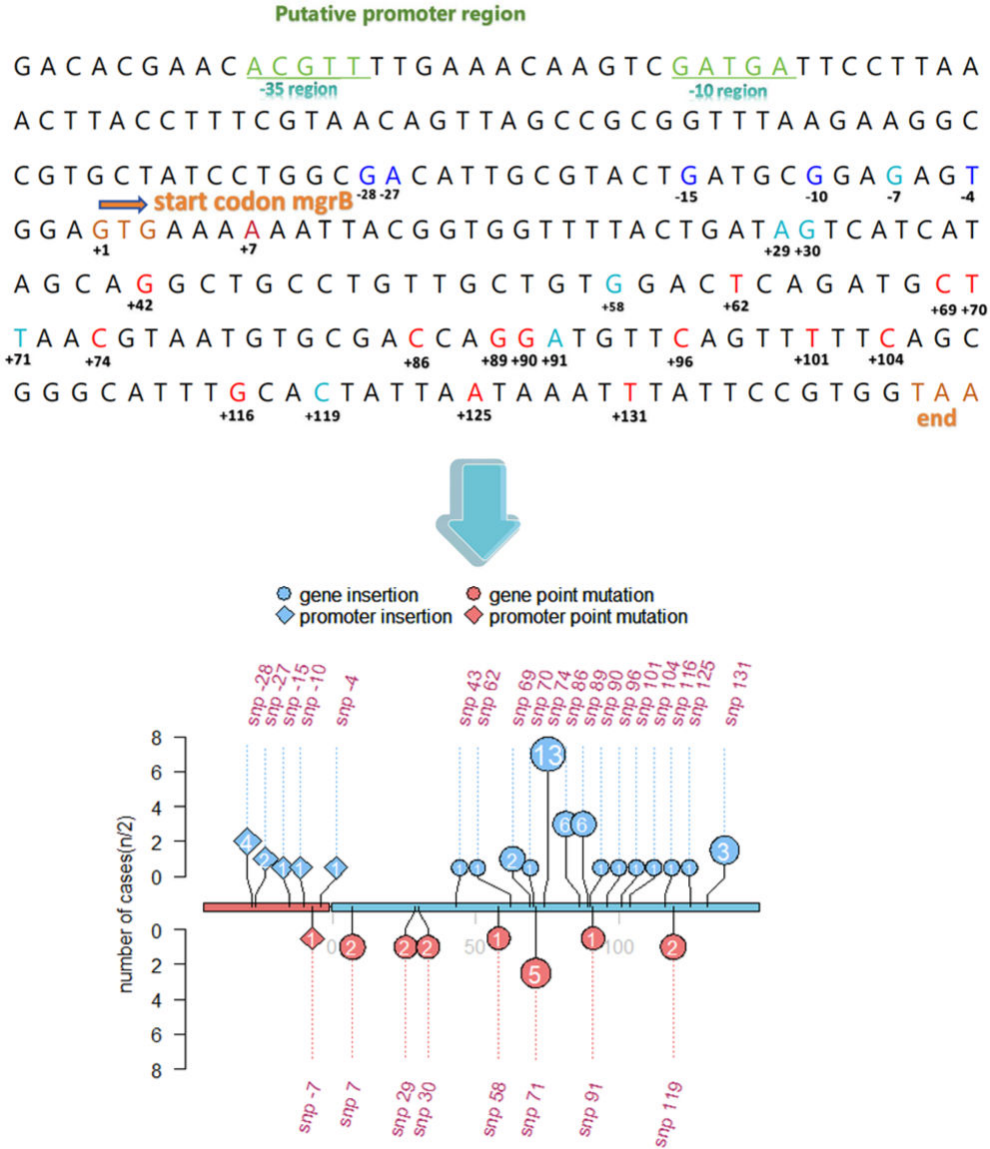
Mutation loci in the *mgrB* gene of 134 strains. In the diagram, *mgrB* sequence and position are shown. Blue color represents insertions in the promoter region. Light blue color indicates insertions within the gene sequence. Red color denotes point mutation in the sequence. In the position display, different shapes distinguish mutation types, and numeric values within shapes indicate the number of strains with mutations at each corresponding locus. Axis features: red segment marks the promoter region; blue segment designates the coding gene region; upper axis area displays insertion mutations; lower axis area shows point mutations; and gene positions are annotated in red numbering. The *Y*-axis represents half of the corresponding quantity (*n*/2).

We screened for chromosomal mutations in the TCS genes (*pmrA*/*pmrB*, *phoP*/*phoQ*) and the negative regulator *mgrB*, all of which are implicated in colistin resistance. No mutations were detected in *phoP*, whereas nine isolates harbored missense mutations in *phoQ*. The most frequently observed alterations were T156S and G90Y, each detected in four isolates, while the remaining isolate carried an I122N mutation. Additionally, one isolate exhibited a partial deletion in the *phoQ* gene. Among the *pmrA*/*pmrB* alterations, *pmrB* mutations were predominant, occurring in 35 isolates. The most common missense mutations were T157P (16/134, 11.9%) and A246T (14/134, 10.4%). Less frequent alterations included deletions spanning nucleotide positions 730–740 (three isolates), and single occurrences of M213L and S203P. Notably, one isolate exhibited concurrent mutations in both *pmrA* (G157T) and *pmrB* (M213L) (Table 1). Mutations in *mgrB* represented the primary genetic mechanism conferring colistin resistance in *K. pneumoniae*, with greater clinical relevance than TCS-related mutations. A total of 72 *mgrB*-inactivated isolates were identified, including 53 insertion events, 11 point mutations, and 8 deletions. Alterations in the promoter region included insertions at positions −28, −27, −15, −10, and −4, as well as a point mutation at position −7 (Fig. 4). The most prevalent insertion sequence (IS) elements were IS*Kpn14* (7.4%) and IS*Kpn26* (8.9%). In addition, IS*903B* (4/134) and IS*Kpn18* (1/134) were also detected. Notably, two isolates carried the rare insertion element IS*Rm4-1*. Furthermore, 20 isolates harbored novel IS elements that could not be characterized. Missense mutations in *mgrB* included G119T, G91A, T71A, G58C, G7T, and A7T (11 isolates in total). Deletion events involved complete gene loss (7/134) or partial deletion (1/134). Interestingly, we identified a unique dual mutation pattern in which both an insertion and a point mutation occurred at position +71. The most frequent insertion site was position +74, observed in 13 of 53 insertion cases (24.5%).

## DISCUSSION

*K. pneumoniae* has emerged as one of the most common pathogens associated with hospital-acquired infections. With the increasing prevalence of multidrug-resistant *K. pneumoniae*, colistin and tigecycline have become the last-resort treatment options for combating carbapenem-resistant strains (24). However, the inappropriate or excessive use of colistin has led to the emergence of colistin resistance, now increasingly reported worldwide (25, 26). Between 2014 and 2019, the incidence of colistin resistance rose by 2.4%–3.4% in Europe, 1.2%–2.6% in North America, and 3.3%–6.7% in Asia (27). This growing trend poses a serious therapeutic challenge in the management of CRKP infections.

In this study, we collected 134 CRKP isolates from the First Affiliated Hospital of Zhengzhou University between February 2021 and January 2024. The majority of samples were obtained from patients in the ICU and the lung transplant department, with most isolates derived from sputum and bronchoalveolar lavage fluid. Consistent with previous reports (28, 29), ST11-K64 KPC-2-producing clones remained the predominant carbapenemase-producing CRKP lineage in our setting. Among the 134 isolates, 127 (94.8%) harbored the *bla*_KPC-2_ gene. Clinical treatment data showed a strong preference for tigecycline, which was prescribed to 89 patients. Notably, most isolates remained susceptible to tigecycline, even in patients with prior tigecycline exposure.

Although many studies have attributed the emergence of colistin resistance to selective drug pressure, colistin remained in use for treating approximately half of the infections in this cohort (30, 31). Therefore, novel therapeutic strategies and alternative antibiotics need to be further explored for the treatment of Col^r^-CRKP. Previous evidence suggests that Col^r^-CRKP isolates may retain susceptibility to gentamicin compared to colistin-susceptible *K. pneumoniae* strains (32). Additionally, ceftazidime-avibactam represents a promising treatment option for infections caused by Col^r^-CRKP (27).

We analyzed the antimicrobial susceptibility profiles of the collected Col^r^-CRKP isolates and found that resistance to meropenem (MEM) and imipenem (IPM) was alarmingly high, reaching 94.7%. In contrast, most isolates remained susceptible to tigecycline. According to clinical medication records, although colistin therapy had been administered to the majority of patients, 48.5% (59/134) had no documented history of colistin exposure. This finding implies that colistin resistance in nearly half of the isolates may have arisen independently of direct drug selection pressure, suggesting alternative drivers of resistance evolution, such as environmental reservoirs or spontaneous chromosomal mutations. While tigecycline demonstrated retained *in vitro* activity, the reliance on a single agent poses substantial risks in the clinical management of Col^r^-CRKP. Consequently, there is an urgent need to develop and implement novel therapeutic strategies or optimized combination regimens. Recent studies have proposed various synergistic antibiotic combinations as potentially effective approaches for treating these highly resistant infections (33, 34), which may help mitigate both the incidence and clinical burden associated with Col^r^-CRKP.

Over the 3-year surveillance period, we collected a large number of CRKP isolates and performed a comprehensive analysis of their colistin resistance determinants. Given its clinical and epidemiological importance, the plasmid-mediated colistin resistance gene *mcr*, which facilitates horizontal gene transfer across species, was closely examined. Among all isolates, *mcr* genes were detected in only two strains, harboring *mcr-1.1* and *mcr-8.2*. These *mcr* genes, commonly associated with animal and environmental sources, were rarely observed in Col^r^-CRKP strains circulating among human populations. In contrast, chromosomal mechanisms—particularly mutations in *pmrA*/*pmrB*, *phoP*/*phoQ*, and *mgrB*—played a more substantial role in mediating colistin resistance. To explore the distribution and variation of these genetic determinants, we constructed a phylogenetic tree and categorized isolates into seven distinct clusters (A–G) based on multilocus ST and capsular serotype (K-type). Cluster A, composed of ST11-K25 isolates, contained the majority of tigecycline-resistant strains in our collection. Notably, this cluster demonstrated higher genetic diversity compared to the others, suggesting the need for further investigation into its unique evolutionary dynamics.

In clinical settings, ST11 is typically classified into two major capsular types: K64 and K47 (35). Interestingly, our study also identified ST11-K62 as a frequently occurring lineage, indicating that this genotype may represent a regionally prevalent strain in Henan Province. Phylogenetic analysis further revealed three distinct ST11 sublineages—Clusters B, C, and G—corresponding to K47, K62, and K64 capsular types, respectively. These clusters exhibited similar antimicrobial susceptibility profiles. Although mutations in both *pmrA*/*pmrB* and *phoP*/*phoQ* were observed across clusters, TCS alterations were predominantly identified in *pmrA*/*pmrB*. Notably, only *pmrA*/*pmrB* mutations were detected in Clusters B and C.

Group E consisted of ST15-K19 strains, whereas Group F was composed of ST239-K19 strains. Nearly all isolates within these two groups harbored mutations in either the *mgrB* gene or TCS genes. The key distinction between the two clusters lies in the location of the mutations: *mgrB* disruptions predominated in Group E, while TCS-related mutations were more frequent in Group F.

Interestingly, our findings differ from a previous study that reported colistin resistance in *K. pneumoniae* as primarily driven by the upregulation of the *phoP*/*Q* system (36). In contrast, a greater proportion of our isolates carried mutations in the *pmrA*/*pmrB* genes. This discrepancy may be attributable to differences in sampling scope or geographic distribution. Overall, these observations highlight the complexity of TCS-mediated resistance and underscore the need for more comprehensive studies to elucidate their precise regulatory roles and contributions to colistin resistance.

*MgrB* is a negative feedback regulator of the TCS (36). Alterations in *mgrB*—including insertion of diverse insertion sequences (ISs) and amino acid substitutions—have become the primary mechanisms of colistin resistance in *K. pneumoniae* (37–40). According to one study, 53.7% of colistin-resistant *K. pneumoniae* isolates carried mutated *mgrB* (41), highlighting the clinical relevance of this resistance mechanism. Various mutations in the *mgrB* locus were observed and classified into four categories: insertional inactivation, point substitution, complete deletion, and partial deletion. The prevalence of substitution increased from 18% in 2014 to 50% in 2022, while complete deletions rose from 9% to 30% during the same period. Notably, insertional inactivation remained the most common alteration, with a pooled prevalence of 69% (42). The insertion of IS elements results in the truncation or inactivation of *mgrB*, leading to a loss of its regulatory function (38).

The increasing trend in these recombination events may be attributed to the dissemination of carbapenem-resistance plasmids, which frequently harbor mobile genetic elements such as ISs. In our study, the most common IS elements identified in *mgrB* were IS*Kpn14* (6.9%) and IS*Kpn26* (8.3%), both of which are commonly found on carbapenem-resistant plasmids. We speculate that the spread of these resistance plasmids accelerates the dissemination of IS elements, thereby increasing the likelihood of *mgrB* disruption and colistin resistance. This hypothesis is consistent with our observation that colistin resistance was more prevalent among CRKP isolates.

Most IS insertions occurred at the +74 position, with 13 isolates exhibiting insertions at this site. In addition, IS*Kpn18* (*n* = 1) and IS*903B* (*n* = 4) were also detected. These four IS elements all belong to the IS*5* family, a group commonly associated with *mgrB* disruptions (43). Notably, we also identified a novel insertion sequence, IS*Rm4-1*, in one CRKP isolate. While this IS element has been previously reported in other species, to our knowledge, this is the first study documenting its presence in *K. pneumoniae* (44). Given that IS*Rm4-1* is inserted within the coding region of *mgrB* at position +71 and disrupts or inactivates this negative regulator, it suggests that IS*Rm4-1* may exhibit mobilization dynamics similar to IS*5* family elements and could play a previously unrecognized role in the adaptive evolution of antibiotic resistance under selective pressure.

To enhance the robustness of our findings, we conducted statistical analyses comparing resistance determinants across clone clusters. The prevalence of *mgrB* inactivation was significantly higher in ST11-K64 isolates (*P* < 0.01), while TCS mutations were more frequently observed in non-ST11 lineages (*P* < 0.05), suggesting possible lineage-specific resistance evolution.

However, this study has several limitations. We did not investigate the potential for horizontal transmission of Col^r^-CRKP, despite previous reports indicating that person-to-person spread—even in the absence of direct colistin exposure—may represent a significant route of dissemination. Although we identified novel insertion sequences, their precise classification and functional roles remain uncharacterized. Additionally, although ceftazidime-avibactam has been shown in prior studies to be effective against CRKP, we did not perform antimicrobial susceptibility testing for this agent. Moreover, one isolate in our study, PBR144, exhibited high-level colistin resistance despite the absence of known resistance determinants such as *mcr* genes or chromosomal mutations, suggesting the involvement of alternative or previously unidentified resistance mechanisms that merit further investigation.

In conclusion, we performed a comprehensive whole-genome analysis of Col^r^-CRKP isolates collected from a tertiary hospital over a 3-year period. Our findings identified *mgrB* inactivation as the predominant genetic mechanism conferring colistin resistance, whereas the prevalence of plasmid-mediated *mcr* genes was relatively low. Notably, we report for the first time the detection of the novel insertion sequence IS*Rm4-1* in *K. pneumoniae*, which was inserted at position +71 of the *mgrB* gene. Given its potential to disrupt the function of this key regulatory gene, IS*Rm4-1* may represent a previously unrecognized contributor to the evolution of resistance under antibiotic selective pressure. However, due to the limited number of isolates and the narrow range of clinical sources, broader epidemiological surveillance and expanded genomic analysis are needed to elucidate the full spectrum of resistance mechanisms and to inform more effective infection control and treatment strategies for combating Col^r^-CRKP.

## Data Availability

All sequencing data of isolates in this study were deposited in the NCBI genome database and organized under BioProject PRJNA1245379.

## SUPPLEMENTAL MATERIAL

Table S1 spectrum.01049-25-s0001.docx Original statistics of antibiotic susceptibility.

ASM does not own the copyrights to Supplemental Material that may be linked to, or accessed through, an article. The authors have granted ASM a non-exclusive, world-wide license to publish the Supplemental Material files. Please contact the corresponding author directly for reuse.
