# Supplementary material for: Molecular characterization of colistin resistance in carbapenem-resistant Klebsiella pneumoniae from a tertiary hospital in China
Source: Microbiol Spectr. 2025 Aug 29;13(10):e01049-25. doi: 10.1128/spectrum.01049-25 (PMC12502562; doi:10.1128/spectrum.01049-25)
Supplement: Table S1 — Original statistics of antibiotic susceptibility. [file spectrum.01049-25-s0001.docx]

Table S1 Antimicrobial susceptibility of 134 strains in the study

| strain | AMK | SAM | ATM | CIP | MNO | TZP | CXM | CRO | CAZ | TOB | LVX | MEM | IPM | COL | TGC |
| --- | --- | --- | --- | --- | --- | --- | --- | --- | --- | --- | --- | --- | --- | --- | --- |
| PBR1 | 64 | 6 | 64 | 4 | 16 | 128 | 6 | 6 | 64 | 16 | 8 | 16 | 16 | 8 | 2 |
| PBR2 | 64 | 32 | 64 | 4 | 16 | 128 | 64 | 6 | 64 | 16 | 8 | 16 | 16 | 16 | 2 |
| PBR3 | 64 | 6 | 64 | 4 | 16 | 128 | 6 | 6 | 64 | 16 | 8 | 16 | 16 | 64 | 4 |
| PBR4 | 64 | 6 | 64 | 4 | 16 | 128 | 6 | 6 | 64 | 16 | 8 | 16 | 16 | 8 | 2 |
| PBR5 | 64 | 6 | 64 | 4 | 16 | 128 | 6 | 6 | 64 | 16 | 8 | 16 | 16 | 32 | 2 |
| PBR6 | 64 | 6 | 64 | 4 | 8 | 128 | 6 | 6 | 64 | 16 | 8 | 16 | 16 | 64 | 2 |
| PBR7 | 64 | 6 | 64 | 4 | 16 | 128 | 6 | 6 | 32 | 16 | 8 | 16 | 16 | 32 | 2 |
| PBR8 | 64 | 6 | 64 | 4 | 16 | 128 | 6 | 6 | 64 | 16 | 8 | 16 | 16 | 16 | 8 |
| PBR9 | 64 | 6 | 64 | 4 | 16 | 128 | 64 | 32 | 32 | 16 | 8 | 16 | 16 | 16 | 16 |
| PBR10 | 64 | 6 | 64 | 4 | 16 | 128 | 6 | 6 | 64 | 16 | 8 | 16 | 16 | 16 | 2 |
| PBR11 | 64 | 6 | 64 | 4 | 4 | 128 | 6 | 6 | 64 | 16 | 8 | 16 | 16 | 64 | 2 |
| PBR12 | 32 | 16/8 | 32 | 4 | 8 | 64/4 | 16 | 32 | 32 | 8 | 8 | 8 | 8 | 16 | 4 |
| PBR13 | 32 | 16/8 | 32 | 4 | 16 | 64/4 | 16 | 32 | 32 | 8 | 8 | 8 | 8 | 1 | 4 |
| PBR14 | 32 | 16/8 | 32 | 4 | 16 | 64/4 | 16 | 32 | 32 | 8 | 8 | 8 | 8 | 1 | 4 |
| PBR15 | 4 | 6 | 1 | 1 | 8 | 4 | 6 | 6 | 2 | 2 | 1 | 0.25 | 2 | 16 | 2 |
| PBR16 | 64 | 6 | 64 | 4 | 16 | 128 | 6 | 6 | 64 | 16 | 8 | 16 | 16 | 16 | 8 |
| PBR17 | 64 | 6 | 64 | 4 | 16 | 128 | 6 | 6 | 64 | 16 | 8 | 16 | 16 | 64 | 4 |
| PBR18 | 64 | 6 | 64 | 4 | 16 | 128 | 6 | 6 | 64 | 16 | 8 | 16 | 16 | 16 | 4 |
| PBR19 | 64 | 6 | 64 | 4 | 16 | 128 | 6 | 6 | 64 | 16 | 8 | 16 | 16 | 64 | 4 |
| PBR20 | 64 | 6 | 64 | 4 | 16 | 128 | 6 | 6 | 64 | 16 | 8 | 16 | 16 | 16 | 2 |
| PBR21 | 64 | 6 | 64 | 4 | 16 | 128 | 6 | 6 | 64 | 16 | 8 | 16 | 16 | 64 | 8 |
| PBR22 | 64 | 6 | 64 | 4 | 16 | 128 | 6 | 6 | 64 | 16 | 8 | 16 | 16 | 128 | 1 |
| PBR23 | 64 | 6 | 64 | 4 | 16 | 128 | 6 | 6 | 64 | 16 | 8 | 16 | 16 | 64 | 8 |
| PBR24 | 64 | 6 | 64 | 4 | 16 | 128 | 6 | 6 | 64 | 16 | 8 | 16 | 16 | 16 | 8 |
| PBR25 | 64 | 6 | 64 | 4 | 16 | 128 | 6 | 6 | 64 | 16 | 8 | 16 | 16 | 64 | 4 |
| PBR26 | 64 | 6 | 64 | 4 | 8 | 128 | 6 | 6 | 64 | 16 | 8 | 16 | 16 | 64 | 4 |
| PBR28 | 2 | 32 | 64 | 4 | 4 | 128 | 6 | 6 | 64 | 1 | 8 | 16 | 16 | 32 | 2 |
| PBR29 | 64 | 6 | 64 | 4 | 4 | 128 | 6 | 6 | 64 | 16 | 8 | 16 | 16 | 16 | 2 |
| PBR30 | 64 | 6 | 64 | 4 | 16 | 128 | 6 | 6 | 64 | 16 | 8 | 16 | 16 | 32 | 2 |
| PBR31 | 64 | 6 | 64 | 4 | 16 | 128 | 6 | 6 | 64 | 16 | 8 | 16 | 16 | 32 | 8 |
| PBR32 | 64 | 6 | 64 | 4 | 16 | 128 | 64 | 6 | 64 | 16 | 8 | 16 | 16 | 16 | 8 |
| PBR34 | 64 | 6 | 64 | 4 | 16 | 128 | 6 | 6 | 64 | 16 | 8 | 16 | 16 | 16 | 2 |
| PBR35 | 64 | 6 | 64 | 4 | 1 | 128 | 6 | 6 | 64 | 16 | 8 | 16 | 16 | 16 | 0.5 |
| PBR36 | 64 | 6 | 64 | 4 | 4 | 128 | 6 | 6 | 64 | 16 | 8 | 16 | 16 | 16 | 2 |
| PBR37 | 64 | 6 | 64 | 4 | 4 | 128 | 6 | 6 | 64 | 16 | 8 | 16 | 16 | 64 | 2 |
| PBR38 | 64 | 6 | 64 | 4 | 8 | 128 | 14 | 6 | 64 | 16 | 8 | 16 | 16 | 64 | 2 |
| PBR39 | 64 | 6 | 64 | 4 | 16 | 128 | 6 | 6 | 64 | 16 | 8 | 16 | 16 | 128 | 4 |
| PBR40 | 64 | 6 | 64 | 4 | 16 | 128 | 6 | 6 | 64 | 16 | 8 | 16 | 16 | 16 | 2 |
| PBR41 | 64 | 6 | 64 | 4 | 16 | 128 | 6 | 6 | 64 | 16 | 8 | 16 | 16 | 64 | 2 |
| PBR42 | 2 | 6 | 64 | 4 | 16 | 128 | 6 | 6 | 64 | 16 | 8 | 16 | 8 | 8 | 2 |
| PBR43 | 64 | 6 | 64 | 4 | 16 | 128 | 6 | 6 | 64 | 16 | 8 | 16 | 16 | 128 | 2 |
| PBR44 | 64 | 6 | 64 | 4 | 16 | 128 | 6 | 6 | 64 | 16 | 8 | 16 | 16 | 32 | 2 |
| PBR45 | 64 | 6 | 64 | 4 | 4 | 128 | 6 | 6 | 64 | 16 | 8 | 16 | 16 | 16 | 2 |
| PBR46 | 64 | 6 | 64 | 4 | 16 | 128 | 6 | 6 | 64 | 16 | 8 | 16 | 16 | 64 | 1 |
| PBR47 | 64 | 6 | 64 | 4 | 16 | 128 | 6 | 6 | 64 | 16 | 8 | 16 | 16 | 128 | 4 |
| PBR48 | 64 | 6 | 64 | 4 | 16 | 128 | 6 | 6 | 64 | 16 | 8 | 16 | 16 | 16 | 4 |
| PBR50 | 64 | 6 | 64 | 4 | 8 | 128 | 6 | 6 | 64 | 16 | 8 | 16 | 16 | 64 | 2 |
| PBR51 | 64 | 6 | 64 | 4 | 16 | 128 | 6 | 6 | 64 | 16 | 8 | 16 | 16 | 64 | 2 |
| PBR52 | 64 | 6 | 64 | 4 | 16 | 128 | 6 | 6 | 64 | 16 | 8 | 16 | 16 | 64 | 2 |
| PBR53 | 64 | 32 | 64 | 4 | 16 | 128 | 6 | 6 | 64 | 16 | 8 | 16 | 16 | 64 | 2 |
| PBR56 | 64 | 6 | 64 | 4 | 16 | 128 | 6 | 6 | 6 | 16 | 8 | 16 | 16 | 64 | 8 |
| PBR57 | 64 | 6 | 64 | 4 | 4 | 128 | 6 | 6 | 64 | 16 | 8 | 16 | 16 | 8 | 2 |
| PBR58 | 64 | 6 | 64 | 4 | 16 | 128 | 6 | 6 | 64 | 16 | 8 | 16 | 16 | 32 | 2 |
| PBR59 | 64 | 6 | 64 | 4 | 16 | 128 | 6 | 6 | 64 | 16 | 8 | 16 | 16 | 16 | 2 |
| PBR60 | 18 | 6 | 64 | 4 | 6 | 128 | 6 | 6 | 64 | 8 | ‘-- | 16 | 16 | 64 | 4 |
| PBR62 | 64 | 6 | 64 | 4 | 16 | 128 | 6 | 6 | 64 | 16 | 8 | 16 | 16 | 16 | 2 |
| PBR63 | 64 | 6 | 64 | 4 | 16 | 128 | 6 | 6 | 64 | 16 | 8 | 16 | 16 | 16 | 2 |
| PBR67 | 64 | 64 | 32 | 16 | 16 | 128 | 6 | 6 | 64 | 16 | 8 | 16 | 16 | 16 | 2 |
| PBR68 | 64 | 6 | 64 | 4 | 16 | 128 | 64 | 6 | 64 | 16 | 8 | 16 | 16 | 16 | 2 |
| PBR69 | 64 | 6 | 64 | 4 | 8 | 128 | 64 | 6 | 64 | 16 | 8 | 16 | 16 | 16 | 2 |
| PBR70 | 64 | 6 | 64 | 4 | 16 | 128 | 6 | 6 | 64 | 16 | 8 | 16 | 16 | 64 | 1 |
| PBR71 | 64 | 6 | 64 | 4 | 16 | 128 | 6 | 6 | 64 | 16 | 8 | 16 | 16 | 64 | 1 |
| PBR72 | 4 | 6 | 64 | 4 | 16 | 128 | 6 | 6 | 64 | 16 | 8 | 16 | 16 | 16 | 2 |
| PBR73 | 64 | 64 | 2 | 16 | 8 | 128 | 64 | 6 | 64 | 16 | 8 | 4 | 16 | 16 | 0.5 |
| PBR74 | 64 | 6 | 64 | 4 | 16 | 128 | 6 | 6 | 64 | 16 | 8 | 16 | 16 | 16 | 2 |
| PBR75 | 64 | 6 | 64 | 4 | 16 | 128 | 6 | 6 | 64 | 16 | 8 | 16 | 16 | 64 | 2 |
| PBR76 | 64 | 6 | 64 | 4 | 16 | 128 | 6 | 6 | 64 | 16 | 8 | 16 | 16 | 64 | 2 |
| PBR78 | 64 | 6 | 64 | 4 | 4 | 128 | 6 | 6 | 64 | 16 | 8 | 16 | 16 | 32 | 2 |
| PBR79 | 64 | 32 | 64 | 4 | 8 | 128 | 6 | 6 | 64 | 16 | 8 | 16 | 16 | 16 | 2 |
| PBR81 | 64 | 6 | 64 | 4 | 16 | 128 | 6 | 6 | 64 | 16 | 8 | 16 | 16 | 16 | 2 |
| PBR84 | 64 | 6 | 64 | 4 | 16 | 128 | 6 | 6 | 64 | 16 | 8 | 16 | 16 | 64 | 2 |
| PBR85 | 64 | 6 | 64 | 4 | 16 | 128 | 6 | 6 | 64 | 16 | 8 | 16 | 16 | 128 | 32 |
| PBR86 | 2 | 14 | 1 | 4 | 8 | 4 | 6 | 6 | 0.5 | 8 | 8 | 0.25 | 0.25 | 16 | 1 |
| PBR87 | 64 | 6 | 64 | 4 | 16 | 128 | 6 | 6 | 64 | 16 | 8 | 16 | 16 | 64 | 2 |
| PBR88 | 64 | 6 | 64 | 4 | 16 | 128 | 6 | 6 | 64 | 16 | 8 | 16 | 16 | 16 | 2 |
| PBR89 | 64 | 6 | 64 | 4 | 16 | 128 | 6 | 6 | 64 | 16 | 8 | 16 | 16 | 16 | 2 |
| PBR90 | 64 | 6 | 64 | 4 | 16 | 128 | 6 | 6 | 64 | 16 | 8 | 16 | 16 | 128 | 2 |
| PBR92 | 64 | 6 | 64 | 4 | 16 | 128 | 6 | 6 | 64 | 16 | 8 | 16 | 16 | 128 | 2 |
| PBR95 | 2 | 6 | 64 | 4 | 16 | 128 | 6 | 6 | 64 | 1 | 8 | 16 | 16 | 64 | 2 |
| PBR96 | 2 | 6 | 64 | 4 | 16 | 128 | 6 | 6 | 32 | 1 | 8 | 16 | 16 | 64 | 2 |
| PBR97 | 2 | 6 | 64 | 4 | 16 | 128 | 6 | 6 | 64 | 1 | 8 | 16 | 16 | 64 | 2 |
| PBR98 | 64 | 6 | 64 | 4 | 16 | 128 | 6 | 6 | 64 | 16 | 8 | 16 | 16 | 32 | 2 |
| PBR101 | 4 | 6 | 64 | 4 | 16 | 128 | 6 | 6 | 64 | 16 | 8 | 16 | 16 | 64 | 2 |
| PBR102 | 2 | 6 | 64 | 4 | 16 | 128 | 6 | 6 | 64 | 1 | 8 | 16 | 16 | 16 | 2 |
| PBR103 | 64 | 6 | 64 | 4 | 16 | 128 | 6 | 6 | 64 | 16 | 8 | 16 | 16 | 64 | 2 |
| PBR109 | 2 | 6 | 64 | 4 | 16 | 128 | 6 | 6 | 64 | 8 | 8 | 16 | 16 | 32 | 2 |
| PBR110 | 2 | 6 | 64 | 4 | 8 | 128 | 6 | 6 | 64 | 1 | 8 | 16 | 16 | 64 | 2 |
| PBR111 | 2 | 6 | 64 | 4 | 8 | 128 | 6 | 6 | 64 | 1 | 8 | 16 | 16 | 64 | 2 |
| PBR112 | 2 | 6 | 64 | 4 | 8 | 128 | 6 | 6 | 64 | 1 | 8 | 16 | 16 | 64 | 2 |
| PBR113 | 2 | 6 | 64 | 4 | 16 | 128 | 6 | 6 | 64 | 1 | 8 | 16 | 16 | 64 | 2 |
| PBR116 | 64 | 6 | 64 | 4 | 16 | 128 | 6 | 6 | 64 | 16 | 8 | 16 | 16 | 16 | 4 |
| PBR117 | 32 | 16/8 | 32 | 4 | 8 | 64/4 | 16 | 32 | 32 | 8 | 8 | 8 | 8 | 128 | 4 |
| PBR119 | 2 | 6 | 64 | 4 | 16 | 128 | 6 | 6 | 64 | 1 | 8 | 16 | 16 | 64 | 2 |
| PBR120 | 2 | 6 | 64 | 4 | 8 | 128 | 6 | 6 | 64 | 1 | 8 | 16 | 16 | 64 | 2 |
| PBR121 | 2 | 6 | 64 | 4 | 16 | 128 | 6 | 6 | 64 | 1 | 8 | 16 | 16 | 64 | 2 |
| PBR122 | 2 | 6 | 64 | 4 | 16 | 128 | 6 | 6 | 64 | 8 | 8 | 16 | 16 | 16 | 2 |
| PBR126 | 2 | 6 | 64 | 4 | 16 | 128 | 6 | 6 | 64 | 1 | 8 | 16 | 16 | 64 | 2 |
| PBR127 | 2 | 6 | 64 | 4 | 2 | 128 | 6 | 6 | 64 | 1 | 8 | 16 | 0.25 | 128 | 0.5 |
| PBR128 | 2 | 6 | 64 | 4 | 2 | 128 | 6 | 6 | 64 | 1 | 8 | 16 | 0.25 | 16 | 0.5 |
| PBR130 | 64 | 6 | 64 | 4 | 16 | 128 | 6 | 6 | 64 | 16 | 8 | 16 | 16 | 16 | 4 |
| PBR131 | 2 | 6 | 64 | 4 | 8 | 128 | 6 | 6 | 32 | 8 | 8 | 16 | 16 | 16 | 2 |
| PBR132 | 2 | 26 | 1 | 0.25 | 16 | 4 | 38 | 41 | 0.12 | 1 | 0.12 | 0.25 | 2 | 16 | 4 |
| PBR133 | 2 | 6 | 64 | 4 | 8 | 128 | 6 | 6 | 32 | 8 | 8 | 16 | 16 | 16 | 2 |
| PBR134 | 2 | 6 | 64 | 4 | 16 | 128 | 6 | 6 | 64 | 1 | 8 | 16 | 16 | 64 | 2 |
| PBR135 | 2 | 6 | 64 | 4 | 16 | 128 | 6 | 6 | 64 | 1 | 8 | 16 | 16 | 16 | 2 |
| PBR136 | 64 | 6 | 64 | 4 | 16 | 128 | 6 | 6 | 64 | 16 | 8 | 16 | 16 | 16 | 2 |
| PBR137 | 64 | 6 | 64 | 4 | 16 | 128 | 6 | 6 | 64 | 16 | 8 | 16 | 16 | 16 | 2 |
| PBR138 | 64 | 6 | 64 | 4 | 16 | 128 | 6 | 6 | 64 | 16 | 8 | 16 | 16 | 16 | 2 |
| PBR139 | 64 | 6 | 64 | 4 | 16 | 128 | 6 | 64 | 64 | 16 | 16 | 16 | 16 | 16 | 2 |
| PBR140 | 64 | 6 | 64 | 4 | 16 | 128 | 6 | 6 | 64 | 16 | 8 | 16 | 16 | 16 | 2 |
| PBR141 | 2 | 6 | 64 | 4 | 16 | 128 | 6 | 6 | 64 | 8 | 8 | 16 | 16 | 64 | 2 |
| PBR142 | 64 | 6 | 64 | 4 | 16 | 128 | 6 | 6 | 64 | 16 | 8 | 16 | 16 | 16 | 2 |
| PBR143 | 2 | 10 | 1 | 0.25 | 16 | 16 | 10 | 26 | 0.5 | 1 | 1 | 0.25 | 0.25 | 4 | 2 |
| PBR144 | 2 | 6 | 4 | 4 | 16 | 16 | 6 | 6 | 2 | 2 | 8 | 0.25 | 0.25 | 512 | 2 |
| PBR145 | 64 | 64 | 16 | 4 | 16 | 128 | 6 | 6 | 64 | 16 | 8 | 16 | 16 | 16 | 2 |
| PBR146 | 64 | 64 | 16 | 4 | 16 | 128 | 6 | 6 | 64 | 16 | 8 | 16 | 16 | 16 | 2 |
| PBR147 | 64 | 64 | 16 | 4 | 16 | 128 | 6 | 6 | 64 | 16 | 8 | 16 | 16 | 16 | 8 |
| PBR148 | 64 | 64 | 16 | 4 | 16 | 128 | 6 | 6 | 64 | 16 | 8 | 16 | 16 | 128 | 2 |
| PBR149 | 64 | 64 | 8 | 4 | 16 | 128 | 6 | 6 | 64 | 16 | 8 | 16 | 16 | 128 | 2 |
| PBR150 | 64 | 64 | 16 | 4 | 16 | 128 | 6 | 6 | 64 | 16 | 8 | 16 | 16 | 128 | 2 |
| PBR151 | 2 | 6 | 2 | 4 | 16 | 16 | 6 | 6 | 2 | 2 | 4 | 0.25 | 0.25 | 512 | 2 |
| PBR152 | 2 | 6 | 2 | 2 | 16 | 16 | 6 | 6 | 1 | 2 | 4 | 0.25 | 0.25 | 64 | 2 |
| PBR153 | 64 | 64 | 16 | 4 | 16 | 128 | 6 | 6 | 64 | 16 | 8 | 16 | 16 | 32 | 8 |
| PBR155 | 64 | 64 | 16 | 4 | 16 | 128 | 6 | 6 | 64 | 16 | 8 | 16 | 16 | 64 | 2 |
| PBR156 | 64 | 64 | 16 | 4 | 16 | 128 | 6 | 6 | 64 | 16 | 8 | 16 | 16 | 128 | 2 |
| PBR157 | 2 | 6 | 64 | 4 | 16 | 128 | 6 | 6 | 64 | 1 | 8 | 16 | 16 | 128 | 2 |
| PBR159 | 64 | 6 | 64 | 4 | 16 | 128 | 6 | 6 | 64 | 16 | 8 | 16 | 16 | 16 | 2 |
| PBR160 | 64 | 64 | 4 | 4 | 8 | 128 | 6 | 6 | 32 | 16 | 8 | 16 | 16 | 128 | 4 |
| PBR161 | 64 | 64 | 16 | 4 | 16 | 128 | 6 | 6 | 64 | 16 | 8 | 16 | 16 | 64 | 2 |
| PBR162 | 2 | 64 | 2 | 4 | 1 | 128 | 6 | 6 | 64 | 1 | 8 | 16 | 16 | 128 | 0.5 |
| PBR163 | 64 | 64 | 16 | 4 | 16 | 128 | 6 | 6 | 64 | 16 | 8 | 16 | 16 | 64 | 2 |
| PBR164 | 6 | 6 | 64 | 4 | 8 | 128 | 7 | 7 | 32 | 8 | 8 | 16 | 16 | 16 | 2 |
| PBR165 | 64 | 6 | 64 | 4 | 16 | 128 | 6 | 6 | 6 | 16 | 8 | 16 | 16 | 2 | 2 |
| PBR166 | 2 | 64 | 4 | 4 | 8 | 128 | 6 | 6 | 32 | 8 | 8 | 16 | 16 | 16 | 2 |

Antimicrobial agents are abbreviated as follows: AMK: Amikacin; SAM: Ampicillin/Sulbactam; ATM: Aztreonam; CIP: Ciprofloxacin; MNO: Minocycline; TZP: Piperacillin/Tazobactam; CXM: Cefuroxime; CRO: Ceftriaxone; CAZ: Ceftazidime; TOB: Tobramycin; LVX: Levofloxacin; MEM: Meropenem; IPM: Imipenem; COL: Colistin; TGC: Tigecycline;
